# Single-cell technologies in multiple myeloma: new insights into disease pathogenesis and translational implications

**DOI:** 10.1186/s40364-023-00502-8

**Published:** 2023-05-31

**Authors:** Mengping Chen, Jinxing Jiang, Jian Hou

**Affiliations:** Department of Hematology, Ren Ji Hospital, Shanghai Jiao Tong University School of Medicine, Shanghai, 200127 China

**Keywords:** Multiple myeloma, Single-cell sequencing, Tumor heterogeneity, Tumor microenvironment, Clinical translation

## Abstract

Multiple myeloma (MM) is a hematological malignancy characterized by clonal proliferation of plasma cells. Although therapeutic advances have been made to improve clinical outcomes and to prolong patients’ survival in the past two decades, MM remains largely incurable. Single-cell sequencing (SCS) is a powerful method to dissect the cellular and molecular landscape at single-cell resolution, instead of providing averaged results. The application of single-cell technologies promises to address outstanding questions in myeloma biology and has revolutionized our understanding of the inter- and intra-tumor heterogeneity, tumor microenvironment, and mechanisms of therapeutic resistance in MM. In this review, we summarize the recently developed SCS methodologies and latest MM research progress achieved by single-cell profiling, including information regarding the cancer and immune cell landscapes, tumor heterogeneities, underlying mechanisms and biomarkers associated with therapeutic response and resistance. We also discuss future directions of applying transformative SCS approaches with contribution to clinical translation.

## Introduction

MM is a hematological cancer characterized by uncontrolled proliferation of malignant plasma cells (PCs) in the bone marrow (BM) which remains largely incurable [1]. MM accounts for around 10% of hematological cancers with about 155,688 patients to be diagnosed worldwide per year [2]. The survival in MM patients has significantly improved over the past decade [3], owing to the introduction of effective novel regimens including proteasome inhibitors (PIs), monoclonal antibodies and immunomodulatory drugs (IMiDs). However, these patients ultimately develop disease relapse, thus further treatment is required [4]. Clonal evolution and diversity of MM cells and BM microenvironment (BME) changes are the major causes of the disease relapse and poor response rate, and these diverse alterations pose both challenges and opportunities for myeloma therapy [5, 6]. With the development of the high throughput next generation sequencing (NGS), we have gained greater insights into MM biology by exploring its intricate genomic landscape [7–10]. Integrated examination of bulk genomic, transcriptomic and exome sequencing has provided valuable information regarding disease drivers including translocations, copy number alterations, somatic mutations, and altered gene expression. Besides, at cellular level, active interactions between myeloma cells and their microenvironment, including bone marrow stromal cells (BMSCs) and immune cells have been extensively discussed [11, 12]. The molecular mechanisms underlying the progression of this malignancy are driven by signals coming from the BME and immune surveillance failure [13, 14]. An increasing number of evidences suggests that the impairments of immunological processes contribute to myeloma evasion from immune surveillance and resistance to effector cells mediated cytotoxicity, resulting in an immune suppressive BME of myeloma [15, 16].

**Fig. 1:**
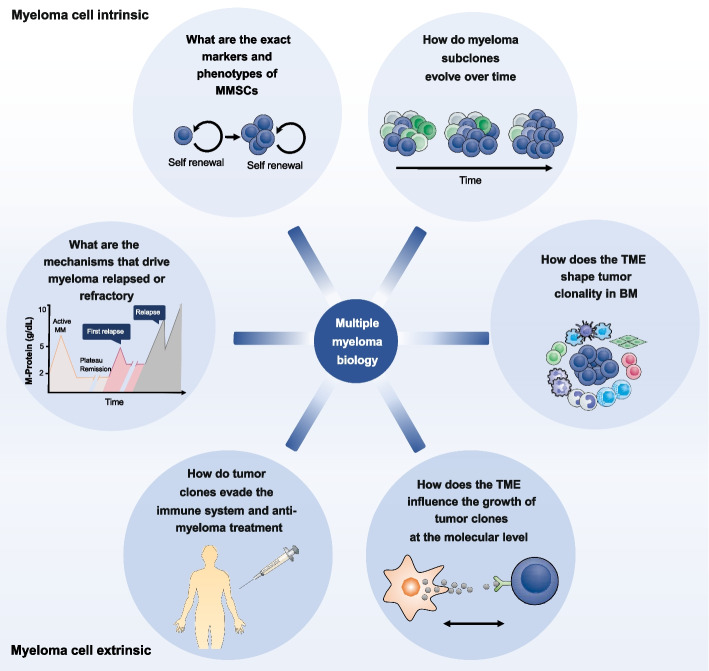
Unresolved questions in the field of myeloma biology research. Studying MM biology in both myeloma intrinsic and extrinsic contexts will advance understanding of how the complex cross-talk between myeloma cells and surrounding non-cancer cells results in the successful growth of malignant subclones, with impacts on clonal revolution and resistance to therapies. MM: multiple myeloma; MMSCs: multiple myeloma stem cells; TME: tumor microenvironment; BM: bone marrow

Despite the impressive progress in understanding the molecular pathogenesis of MM, in developing new therapies and improving in transplant technology [17], many important questions have yet to be addressed, leading to several confronting issues and challenges in the MM field (Fig. 1). One of the key questions is that the identity of the MM cell origin remains controversial. Researchers revealed that cancer stem cell might be responsible for the development of MM, although there is ongoing debate regarding the identity of the MM stem cells (MMSCs) [18–20]. Several markers such as side population (SP) [21], ALDH1^+^ [22] and CD24^+^ [23] have been used to identify MMSCs. Verifying the origin of myeloma cells represents a significant effort to achieve effective cancer treatment. Clonal evolution is also a key topic in MM research field which drives tumor progression, chemoresistance and relapse in myeloma [9, 10, 24–26]. NGS studies have demonstrated different types of clonal changes over the course of disease, which are categorized into stable, linear, and branching evolution of myeloma clones [27, 28]. The major queries are how these subclones arise and how they are selected. As a consequence of current bulk sequencing methods, the answer is certainly equivocal and more powerful technologies are needed. BM myeloma cells are highly dependent on neighboring cell signals for survival which allows them to grow and proliferate. The BME consists of multiple cellular compartments, including mesenchymal stromal cells (MSCs), immune cells, endothelial cells, osteoblasts and osteoclasts, creating a distinct milieu that supports immune escape and promotes progression of myeloma cells. The mechanisms by which the BM niche contributes to MM pathogenesis remain largely unexplored. Despite landmark therapeutic advancements to treat MM, one of the major challenges remains that the majority of patients eventually relapse and become refractory to anti-myeloma drugs. Additionally, there are no reliable biomarkers available for the accurate prediction of responses to specific drug classes, limiting our ability to select a more personalized treatment strategy. Therefore, it is becoming increasingly obvious that there is a prominent need to develop and exploit more powerful and precise approaches to fully dissect the molecular and cellular landscape during disease pathogenesis, and to discover new biomarkers for predicting drug efficacy or resistance, thus providing opportunities for precision medicine of MM.

Traditional microarrays and NGS assays require bulk DNA or RNA from large number of cells, and they are limited to providing average information of a population of cells. Furthermore, rare cell populations or unique cellular states could be critical in tumor transformation and pathogenesis, such as cancer stem cells (CSCs) or immune cell subsets, which might not be detected in bulk analyses [29]. The advent of single-cell sequencing (SCS) has overcome these limitations through revealing the genomic/transcriptomic profile of each cell within given samples at high resolution and throughput [30–32]. SCS has been widely applied in the field of myeloma research, providing the analysis of cellular heterogeneity [33, 34], identifying new cell subtypes [35], distinct cellular states [36], and elucidating dynamic cellular transitions during tumour evolution and microenvironment remodeling [37–39]. These multi-faceted, high-dimensional dissections at genomic, epigenomic, transcriptomic, and proteomic levels in tumour cells and the related immune or stromal cells allow the in-depth characterization of cancer biology, the intricate interactions between cancer cells and surrounding compartments in BME, and details of the clonal evolution in each MM tumour. In this review, we summarize recent progress in the SCS workflow and techniques that have been used in MM research and further discuss the findings of clonal revolution, cancer cellular heterogeneity, stromal cells and immune microenvironment explored by SCS. We further discuss the growing applications of single-cell approaches for answering important research questions and their implications in clinical translation.

## Emerging single-cell sequencing technologies

**Fig. 2:**
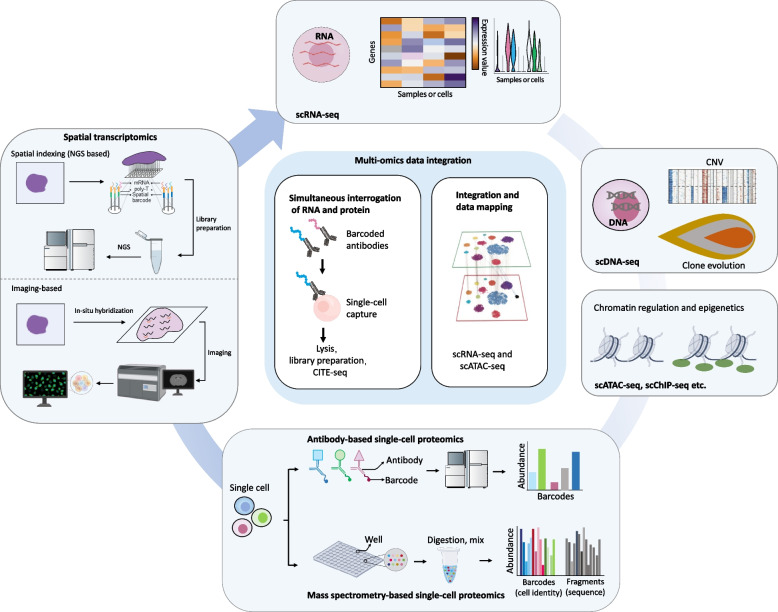
Single-cell approaches utilized in disease research. Single-cell approaches are developed to profile the genome, epigenome, transcriptome/spatial transcriptome and proteome, and single-cell multi-omics approaches are built by combining different SCS methods, which have been widely used in disease research. SCS: single-cell sequencing; scRNA-seq: single-cell RNA sequencing; scDNA-seq: single-cell DNA sequencing; CNV: copy number variation; scATAC-seq: single-cell assay for transposase-accessible chromatin by sequencing; scChIP-seq: single-cell chromatin immunoprecipitation followed by sequencing; NGS: next generation sequencing; CITE-seq: cellular indexing of transcriptomes and epitopes by sequencing

Breakthroughs in single-cell capture, sequencing technologies, and analytical bioinformatics have led to rapid progress in SCS analysis methodologies which have been reviewed extensively elsewhere [40–43]. The success of single-cell approaches benefits from concurrent improvements in methodological pipeline and analytics, such as the isolation of single cells, high-dimensional reduction, unsupervised clustering, evolutionary modelling, multiple datasets integration, lineage tracing and ligand-receptor interaction predicting [43–45]. Single-cell approaches have the unique potential to help answer many important questions that lie in cancer research, including: the roles of cancer and immune cells heterogeneities; the relations between tumor cell clonotype and phenotype; the network of ligand-receptor interactions present in the tumor microenvironment (TME); as well as the spatio-temporal crosstalk between cancer cells and immune populations. With the rapid advancements of SCS technologies, numerous methods have been developed, offering unprecedented opportunities not only to profile DNA, mRNA, chromatin and proteins through different single-cell omics, but also to measure multiple modalities one cell at a time by various multi-omics tools (Table 1). In this part, we will discuss the most important single-cell approaches and platforms that have been utilized in disease research (Fig. 2).

**Table 1:** Technologies for high-throughput single-cell sequencing

Category	Technology	Method	Description	Advantages	Disadvantages	Commercial supplier	Reference
Single- omics	Single-cell transcriptomics	10X Chromium	10X Chromium single cell gene expression	High throughput, high reproducibility,low technical noise, time saving	High cost, high sample requirement	10X Genomics	[46]
		Drop-seq	Droplet-based single cell RNA sequencing	High throughput, low cost, fast	Low cell capture efficiency, low sensitivity	Dolomite Bio	[47]
		inDrop	Droplet-based single cell RNA sequencing	High throughput, low cost	Low mRNA capture efficiency, high error rate	1CellBio	[48]
		Seq-Well	Single-cell RNA-seq with microwells	High throughput, low cost	Low cell capture efficiency	-	[49]
		CytoSeq	Cytometry-based sequencing	High throughput, low cost	Cell size limited (smaller than 20 μm)	BD Rhapsody	[50]
		Smart-seq2	Switching mechanism at the end of the 5′-end of the RNA transcript sequencing	Full-length coverage across transcripts, high sensitivity	High cost, low throughput	Fluidigm C1	[51]
		CEL-Seq2	Single-cell RNA-seq by multiplexed linear amplification	High sensitivity, low cost	Strong 3' preference, low throughput	-	[52]
		sci-RNA-seq	Single-cell combinatorial indexing RNA sequencing	High throughput, minimize perturbation to RNA integrity	Some cell types cannot be defined	-	[53]
		MARS-seq	Massively parallel single-cell RNA sequencing	High throughput	Low sensitivity, high dropout rate	-	[54]
	Single-cell spatial transcriptomics	Spatial indexing	Spatial indexing methods perform hybridization of RNAs to barcoded capture arrays, followed by fragment pooling and NGS	Unbiased, greater coverage, greater field of view, more accessible (typically sequenced using standard NGS machine)	Limited to capture spot resolution, lower depth (per transcript)	10X Visuim, BGI STOmics (Stereo-Seg), AtlasXomics (DBiT-seq)	[55–58]
		Imaging-based	Imaging-based approaches use fluorescent tagging of mRNA molecules in situ and high-resolution fluorescence microscopy to detect mRNA transcripts	Single-cell resolution, greater depth (per transcript), better suited to capture subtype change due to spatial influence	Biased, lower coverage, smaller field of view, more read-out noise, requires more specialized equipment	Vizgen (MERFISH), Spatial Genomics (segFISH), NanoString Technologies (CosMx), 10X Genomics Xenium	[59–61]
	Single-cell genomics	DOP-PCR	degenerate oligonucleotide primed PCR	High throughput	Uneven amplification, low coverage, amplification errors, allele dropout	GenomePlex, Mission Bio Tapestri	[62, 63]
		MDA	multiple displacement amplification	Simplicity, high fidelity, low false positive rate	Amplification bias, allele dropout	Qiagen, REPLI-g, 10X Genomics chromium CNV	[64]
		MALBAC	multiple annealing and looping-based amplification cycles	High uniformity, low amplification bias	Allele dropout	Yikon genomics	[65]
	Single-cell epigenomics	RRBS	reduced representation bisulfite sequencing, detecting DNA modification	Relatively low cost, high coverage of the promoters	Low throughput, low coverage of the genome-wide CpG dinucleotides	-	[66]
		WGBS	whole genome bisulfite sequencing, detecting DNA modification	Low amplification bias, correct assignment of paired-end fragments	Low library complexity	-	[67]
		CGI-seq	genome-wide CpG island methylation sequencing, detecting DNA modification	High efficiency, simplified procedure, good coverage of the CpG islands and no DNA damage	Inconsistent and/or low coverage, Low throughput	-	[68]
		ATAC-seq	assay for transposase accessible chromatin sequencing, detecting chromatin accessibility	High coverage, high sensitivity, high-throughput	Low recovery of DNA fragments	10X Chromium Single Cell ATAC, Bio-Rad SureCell ATAC-Seq	[69–71]
		ChIP-seq	chromatin immunoprecipitation sequencing, detecting histone modification	High resolution, high throughput	Highly dependent on the quality of antibody	Mobidrop	[72]
		scCUT&Tag	Cleavage Under Targets and Tagmentation	Low cell inputs, low cost, profiling protein–DNA interactions, fast	Native conditions are not always suitable, not very well suitable for the analysis of regions of the genome that are silenced or contain heterochromatin	-	[73]
		Drop-ChIP	Droplet-based single-cell chromatin immune-precipitation sequencing, detecting histone modification	High throughput, high specificity	Low coverage	-	[72]
	Single-cell proteomics	Mass spectrometry-based	Mass spectrometry-based single-cell proteomics	Quantify more proteins per cell (1,000 to 1,500), label free analysis is permitted, mature data analytics	Limited throughput to ~ 10 cells per hour per instrument, low sensitivity, destructive method	Standard BioTools	[74]
		Antibody-based	Antibody-based single-cell proteomics	Standard high-throughput, suitable for analysis of cell-surface, cytoplasmic and secreted proteins, cells can be either live or fixed, suitable for sorting live cells	Antibody cross-reactivity, quantify fewer protein per cell	BD Rhapsody	[75]
Multi-omics	Genome + transcriptome	G&T-seq	Genome and transcriptome sequencing	Powerful capability to characterize cellular diversity, high accuracy	Low throughput	-	[76]
		DR-seq	gDNA–mRNA sequencing	minimize the risk of losing (deoxy)ribonucleic acids	Low throughput	-	[77]
		TARGET-seq	Targeted mutation detection and parallel transcriptome characterization	minimize the risk of losing (deoxy)ribonucleic acids	Low throughput	-	[78]
	Transcriptome-DNA methylome	scM&T-seq	Single-cell genome-wide methylome and transcriptome sequencing	Amplified DNA and RNA separately and independently	Does not distinguish between 5mC and 5hmC	-	[79]
	Genome + transcriptome + DNA methylome	scTrio-seq	single-cell triple omics sequencing	Simultaneous analyses of genome, epigenome, and transcriptome in the same single cell	Low throughput	-	[80]
	Transcriptome + chromatin accessibility	sci-CAR	single-cell chromatin accessibility and mRNA-seq	Joint profiling of chromatin accessibility and gene expression	Low cell capture efficiency, limited throughput	-	[81]
		Paired-seq	Single-cell RNA and chromatin accessibility sequencing	Powerful capability to characterize cellular diversity, high accuracy	lower library complexity than stand-alone single-cell and snATAC-seq and RNA-seq	-	[53]
	Transcriptome + proteome	CITE-seq	Cellular indexing of transcriptomes and epitopes by sequencing	Providing additional phenotypic information, high compatibility	Only cell surface protein can be characterized	10X Genomics	[82]
		REAP-seq	RNA expression and protein sequencing assay	Can be used to characterize unknown cellular populations, minimizing steric hindrance	Limited to cell surface proteins	Fluidigm C1	[83]
		RAID	single-cell RNA and immuno-detection	Allow combined analysis of the transcriptome and intracellular (phospho-)proteins from fixed single cells	Not suitable for cell surface proteins	-	[84]
	Transcriptome + DNA–protein interactome	scDam&T-seq	single-cell DNA adenine methyltransferase identification and transcriptome sequencing	Allow combined analysis of the transcriptome and DNA–protein interactions	Limits throughput	-	[85]

*Drop-seq* Droplet-based sequencing, *inDrop* indexing droplets, *CytoSeq* Cytometry-based sequencing, *Smart-seq2* Switching mechanism at the end of the 5′-end of the RNA transcript sequencing 2, *CEL-Seq2* Cell expression by linear amplification and sequencing 2, *sci-RNA-seq* single-cell combinatorial indexing RNA sequencing, *MARS-seq* Massively parallel single-cell RNA sequencing, *DOP-PCR* degenerate oligonucleotide primed-polymerase chain reaction, *MDA* multiple displacement amplification, *MALBAC* multiple annealing and looping-based amplification cycles, *RRBS* reduced representation bisulfite sequencing, *WGBS* whole genome bisulfite sequencing, *CGI-seq* CpG island methylation sequencing, *ATAC-seq* assay for transposase accessible chromatin sequencing, *ChIP-seq* chromatin immunoprecipitation sequencing, *scCUT&Tag* single-cell cleavage under targets and tagmentation, *Drop-ChIP* droplet-based single-cell chromatin immune-precipitation sequencing, *G&T-seq* genome and transcriptome sequencing, *DR-seq* gDNA–mRNA sequencing, *TARGET-seq* targeted mutation detection and parallel transcriptome characterization, *scM&T-seq* single-cell genome-wide methylome and transcriptome sequencing, *scTrio-seq* single-cell triple omics sequencing, *sci-CAR* single-cell combinatorial indexing chromatin accessibility and mRNA sequencing, *Paired-seq* paired RNA and DNA accessibility seq, *CITE-seq* cellular indexing of transcriptomes and epitopes by sequencing, *REAP-seq* RNA expression and protein sequencing assay, *RAID* RNA and immunodetection, *scDam&T-seq* single-cell DNA adenine methyltransferase identification and transcriptome sequencing

### Single-cell transcriptomics

Single-cell RNA sequencing (scRNA-seq) is a procedure that enables non-targeted quantification of transcripts in individual cells [86]. This technique is largely independent of previous biology knowledge, and it allows for the detailed description of tissue subtypes and cell states. Multiple efficient high-throughput protocols for single-cell separation have been developed (for example, the Fluidigm C1 platform [87], droplet microfluidics [46] and microwells [88]). And various scRNA-seq approaches such as Drop-seq [47], Smart-seq2 [89], CEL-seq2 [52], MARS-seq [54], CytoSeq [50], sci-RNA-seq [53] have contributed to the discovery of novel and rare cell types, cellular heterogeneity within complex tissues, and biological mechanisms in healthy and disease conditions. A high-throughput sequencing method for mRNA transcriptomics at a single-cell level was first reported in 2009 [90]. Islam et al. developed single-cell tagged reverse transcription sequencing method in 2011, allowing for detecting mixed cell samples including highly heterogeneous tumor samples on a large scale [91]. In 2012, Smart-seq was introduced to facilitate the measurement of full-length transcripts [51]. By enhancing transcriptomic reading coverage, this technology enables accurate analysis of alternative splicing and detection of single nucleotide polymorphisms (SNP) and other genomic mutations. A modified Smart-seq2 was created later by Picelli et al., which improved accuracy, sensitivity and coverage in full-length [92].

In recent years, a number of droplet-based platforms for high-throughput scRNA sequencing have attracted attention, including 10X Chromium Genomics, inDrop and Drop-seq [46, 47, 93]. These technologies share similar strategies in generating droplets, isolating single cells through on-bead primers with barcodes, and correcting bias by applying unique molecular identifiers (UMIs) [94]. However, these technologies differ in cost, time, cell capture efficiency, and sensitivity due to different manufacturing methods for beads, barcoding, and cDNA amplification [94, 95]. In a recent study comparing 10X Genomics and Smart-seq2 sequencing data from CD45^+^ cell samples [96] showed that Smart-seq2 was able to capture more features within a single cell with high sensitivity, in particular capable to detect those cells with low-abundance transcripts and alternative spliced transcripts [96]. Although 10X Genomics method showed higher dropout rates and noise in lowly expressed genes, this tool can detect more genes owing to its preferable coverage of abundant cells, which therefore enable the detection of rare cell populations. In another report, Smart-Seq2 and 10X Genomics were combined to help elucidate the landscape of immune cells and revealed the dynamic status features in hepatocellular carcinoma [97].

### Single-cell spatial transcriptome

Single-cell spatially resolved transcriptome technologies have been developed and improved rapidly in recent years, with a unique capability of capturing cellular spatial distribution and revealing local networks of intercellular communication acting in situ, which cannot be achieved by scRNA-seq. Although there are numerous spatial transcriptomic (ST) methods, current ST protocols can be generally divided into two major groups according to their detection strategies. Most ST techniques use either a spatial indexing or imaging-based approaches to measure and quantify mRNA molecules in situ. In spatial indexing approaches such as 10X Visium and Slide-seq, barcodes are locally hybridized to RNA molecules, then gene expression profiles are quantified by NGS. This approach relies on polyA hybridization, which pose a challenge in fresh-frozen and formalin-fixed paraffin-embedded (FFPE) tissues as mRNA integrity varies in these samples. Further solution on this issue is desirable as clinical samples are often stored in FFPE blocks. Furthermore, achieving single-cell resolution remains a technical and computational challenge. Imaging-based approaches use fluorescent tagging of mRNA molecules in situ, and high-resolution fluorescence microscopy to detect and differentiate between single mRNA transcripts. These methods can achieve single-cell resolution but are still constrained by limitations on probe design, and low in situ mRNA abundance and degradation. Despite these challenges, ST technologies have added a new dimension to single-cell omics and extensively broadened our understanding of cancer biology.

The ST profiling and temporal lineage tracing enable multi-faceted investigations into the surrounding environment and molecular dynamics within a single cell [31], thus add another layer of tumor heterogeneity which might be critical for disease diagnosis, monitoring and treatment in cancer research [98]. Attaching spatial barcodes allows encoding and retrieving location information of single cells, providing important and useful information in research and disease diagnosis. For instance, by positioning histological sections on spatially barcoded microarrays, researchers were able to visualize and quantify the transcriptome with spatial resolution in tissues of mouse brain and human breast cancer [55]. By using this spatial-resolved method, the researchers found prominent tumor heterogeneity within a tumor biopsy section, reflecting different subclones with varying genes expression patterns located in the same area [55]. In addition, a recent study integrated scRNA-seq and microarray-based spatial transcriptomics data from pancreatic ductal adenocarcinomas (PDAC) samples, and revealed that cancer cells with high expression of the stress module colocalized with inflammatory fibroblasts, which may contribute to therapy resistance [99]. Tumor progression is a complex and dynamic process involving multiple steps evolving from initiation, progression to the emergence of therapeutics resistance [100]. Definition of the molecular and temporal nature during these processes is crucial to understand tumor biology and develop effective therapeutics strategies. Lineage tracing by nuclease-activated editing of ubiquitous sequences (LINNAEUS) is a newly developed tool for lineage tracing and has successfully applied to reconstruct lineage trees in zebrafish [101]. CRISPR-Cas9 coupled single-cell analysis was utilized in a KRAS mutant mouse model to decipher a comprehensive spectrum of cancer cells [101]. This approach improved the sensitivity of low mutation, and showed the ability to illustrate the detailed changing of tumor subtypes, and allow tracking the spread pattern of lung cancer cells [102]. These findings suggest that in patients with KRAS mutations, targeted therapy may be developed and clinical management could be improved through these approaches [102]. Another study combining high-confidence clonal tracing and scRNA-seq led to a detailed dissection of leukemic stem cells, providing novel insights into the understanding of leukemia oncogenesis and therapeutics [103].

### Single-cell genomics

Single-cell genomics aims to extend our understanding of genetics by bringing the research of genomes to the cellular level. Thus, rare and unique mutations and copy-number variations (CNVs) can be detected faithfully to reveal the clonal heterogeneity and evolution, which may be involved in disease processes. Sequencing an entire genome of a cell requires whole-genome amplification (WGA) by three major methods including degenerate oligonucleotide primed PCR (DOP-PCR), multiple displacement amplification (MDA) and multiple annealing and looping based amplification cycles (MALBAC) [104]. DOP-PCR often yields low genome coverage, which is pertinent to the exponential amplification of PCR. MDA is considered to be the most suitable method for detecting single nucleotide variants (SNVs) and insertions/deletions at the genome-wide level, owing to its capacity to amplify the majority of a human genome with a high-fidelity polymerase. MALBAC achieves more accuracy for CNV detection and a low false negative rate for SNV detection [104, 105]. High-throughput single-cell DNA sequencing (scDNA-seq) using different sing-cell isolation strategies have been developed by several commercial platforms [106, 107], thus providing higher scalability, allowing cell selection with lower costs, and offering more flexibility for customized chemistry steps. At present, CNV profiling is one of the most common applications of scDNA-seq. Despite the high throughput (up to 10 K cells) achieved by microdroplets (10X Genomics) and combinatorial indexing for single-cell CNV profiling, these methods face challenges of lower data quality and limited genomic resolution. By contrast, FACS, nanowells and microfluidic platforms utilizing tagmentation chemistry [108, 109] are able to provide much high-quality CNV data at single-cell resolution, but provide modest throughput (hundreds to 1,000 cells). Mutation detection presents another major application of scDNA-seq, which requires in-depth coverage of a certain mutation site. While early studies carried out whole-genome or exome sequencing of single cells [110, 111], high cost prevented these studies from profiling large number of cells. In order to increase throughput and reduce costs, later approaches aimed to profile targeted regions of the genome, such as specific cancer gene panels [112, 113]. A microdroplets approach have been developed and commercialized for scaling up scDNA-seq [107], which conducts PCR amplification of single cells at hundreds of targeted genomic regions. ScDNAseq approaches provide a reliable resolution in examining clonal substructure and reconstructing clonal lineages during cancer evolution in the context of premalignant status, metastasis and drug resistance. Technologies for scDNA-seq are particularly helpful to resolve mutual exclusivity and mutation co-occurrence in different clonal subpopulations, which are difficult to identified by bulk sequencing data [113].

### Single-cell epigenomics

Research into epigenetic regulation at the single-cell level has helped to define epigenetic landscape by profiling DNA modifications, chromatin accessibility and histone modifications. A variety of epigenomic sequencing approaches at single-cell level have been developed, such as single-cell reduced-representation bisulfite sequencing (scRRBS) to measure DNA methylation [114], single-cell chromatin immunoprecipitation followed by sequencing (scChIP-seq) to measure histone modifications [72] and protein-DNA interactions [115], and single-cell assay for transposase accessible chromatin sequencing (scATAC-seq) to measure chromatin accessibility [69, 70, 116]. A major advantage of scATAC-seq compared with scRNA-seq is that it offers greater insights into gene regulation and transcription along with cell lineage and identity information [117]. Transcript-indexed ATAC-seq (T-ATAC-seq) approach was built by combining scATAC-seq with sequencing of the T cell receptor (TCR) repertoire, which allows for studying both the epigenomic state and the TCR specificity simultaneously at the single-cell level [118]. Apart from mapping chromatin accessibility, investigating diverse chromatin modifications may provide further insights into epigenomic states. Recently, a new method named single-cell cleavage under targets and tagmentation (CUT&Tag) technology was established to profile multiple histone modifications and DNA-protein interactions [73]. This novel technology generates high-quality data with ultra-low cell inputs compared to traditional ChIP-seq, and helps to study the comprehensive histone modifications and dynamic regulatory interactions with high throughput and sensitivity within each single cell [73].

For better understanding of genomic organization, in situ genome sequencing (IGS) has been used by researchers for simultaneous in situ imaging and sequencing of the genomes within the same single cell [119]. This process includes the construction of in situ genomics DNA libraries, in situ sequencing of amplicons and spatially localized sequences, amplicon dissociation, PCR and ex situ sequencing of amplicons, revealing the accurate localization of distinct DNA sequences. It is clear that IGS presents a valuable mean of addressing biological questions involving the relationships between genomic architecture and disease [119]. Furthermore, integrated high-resolution multiple annealing and looping-based amplify cycles was applied by another study to analyze transcriptomic dynamics and define the three-dimensional genomic architecture at single-cell level [120]. Using this method, researchers can specifically unravel the roles of transcriptomic and genomic architecture during oncogenesis, as well as the interplay among anatomy, function, transcription, and cell types along with cancer progression [120].

### Single-cell proteomics

Proteins are essential macromolecules that are responsible for the main functional machinery within a cell, including regulations of gene expression, signaling pathway and catalytic reaction. Proteome measurements based on mass spectrometry (MS) have historically been limited to bulk samples containing thousands or millions of cells. The rise of single-cell proteomics leads to an in-depth and unbiased profile of protein expression within single cells. This emerging technology is mainly based on two methods: MS-based method, in which the proteomic content of the cell is digested and analyzed; and antibody-based method, which typically target a certain number of predefined proteins [75]. The use of MS is the basis for detecting and quantifying proteomes, but it is only useful for identifying the most abundant proteins. By improving protein preparation and isolation procedures, researchers have been able to decrease protein loss and perform quantitative proteomics sequences at a single-cell resolution. By combining the principles of MS with flow cytometry, mass cytometry by time of flight (CyTOF) uses metal isotope-labeled antibodies conjugated with specific molecules on the cell surface or inside cells, allowing for the examination of 100 specific proteins in single cells [121]. Based on immunohistochemistry with metal-labeled antibodies and CyTOF, imaging mass cytometry (IMC) has been developed [122]. Through IMC, up to 40 protein markers can be analyzed simultaneously, along with their spatial architecture and interactions, which would be lost with traditional lysis of tissue to single cells [123]. Furthermore, IMC can be performed on paraffin-embedded tissue sections, making it useful for retrospective analysis of patient cohorts with known outcomes, ultimately benefitting individualized medicine. Liquid chromatography-mass spectrometry (LC-MS) is another technology for single-cell proteomics analysis [124], which is a bioanalytical method for analyzing proteins quantitatively and is widely used in drug development and drug toxicology research. Furthermore, a multiplexed single-cell proteomics (SCoPE-MS) method has been developed to increase throughput by multiplexing, and enhance peptide sequence identification using an isobaric carrier [125]. As expected, the application of single-cell proteomic sequencing will revolutionize the pathological investigation, particularly when combined with multi-omics platforms, such as transcriptomic and genomic profiling at the single-cell resolution.

### Single-cell multi-omics

Multi-omics single-cell methods interrogate multiple levels of molecular information from a single cell, such as DNA & RNA, RNA & protein, or three layers combined [126]. By combining these methods, it is feasible to gain deeper insight into genotype-phenotype relationships and how genomics affects gene expression and protein level. For instance, the transcriptome and targeted genomic regions can be simultaneously profiled within a single cell, which allows to provide better coverage of the targeted genomic loci for detecting SNVs, deletion mutations as well as CNVs, which are related to drug resistance in lung cancer [127].Quantification of the transcriptome and DNA-protein interactome simultaneously in a single cell will lead to a better understanding of the transcriptional changes during the process of DNA binding to a specific protein, and such quantification has been applied by scDam&T-seq [128]. Furthermore, a new single-cell method named scM&T-Seq was developed for parallel sequencing of DNA methylome and transcriptome in one cell, enabling in-depth analyses of how epigenetic heterogeneity relates to gene expression output at each locus [79]. Moreover, the transcriptome and proteome can also be delineated simultaneously using several technologies, such as CITE-seq [83], REAP-seq [82] and RAID [84]. In a recent study, an integrative pipeline using 3D imaging and the rapid clearing agent FUnGI were used in breast cancer and demonstrated that tumor clones were significantly reduced during oncogenesis, with the luminal progenitor serving as the key cells of origin [129]. In conjunction with multicolor lineage tracing and molecular analysis, LSR-3D imaging offers crucial visual and spatial information within the tumor to describe biological processes, highlighting the nature of tumors with inherent plasticity. Future developments of these multi-omic SCS methods hold promise for overcoming technical hurdles, thereby allowing their widespread adoption in translational research in the near future.

## Applications of single‐cell sequencing in MM biology and pathogenesis

**Fig. 3:**
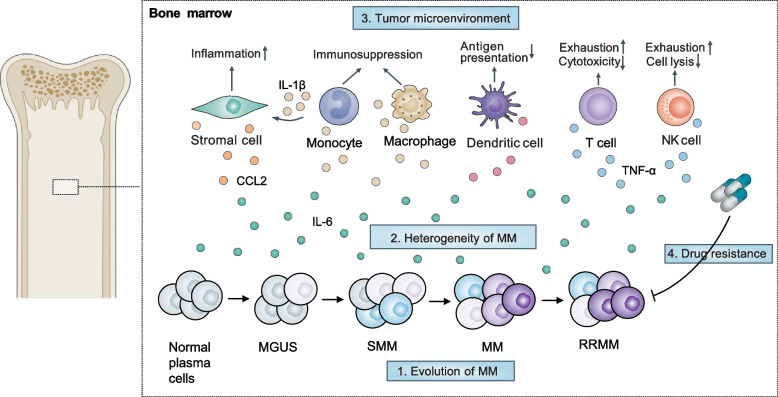
Applications of SCS in myeloma research. SCS has impacted various areas of cancer research and improved our understanding of clonal evolution, tumor heterogeneity, tumor microenvironment, therapeutic resistance in MM, which offers a profound opportunity to improve the diagnosis and identify biomarkers for precision medicine of MM. SCS: single-cell sequencing; MM: multiple myeloma; MGUS: monoclonal gammopathy of undetermined significance; SMM: smoldering multiple myeloma; RRMM: relapsed refractory multiple myeloma

A growing number of studies have begun to investigate the genomic, epigenetic, and transcriptional landscape in MM at the single-cell level, as summarized in Table 2. Herein we summarized the recent SCS studies in MM research and discussed the insights they have provided, which can be roughly categorized into three main aspects: (1) clonal evolution and heterogeneity, (2) reprogramming of the BM microenvironment, (3) response or resistant to anti-myeloma therapy **(**Fig. 3**)**.

**Table 2:** Summary of single-cell studies in MM

Reference	Sample input	Sample size (n)	Methodology	Major findings
Ledergor et al. [33]	BM and PB tumor cells from healthy donors (*n* = 11), MGUS (*n* = 7), SMM (*n* = 6), NDMM (*n* = 12), amyloidosis (*n* = 4)	40	scRNA-seq	Identified inter- and intral-heterogeneity across disease spectrum of MM; detected rare tumour clones at SMM share similar molecular profile with active MM; characteristics of single CTCs reflect the transcriptional status of the matched BMPCs
Khoo et al. [130]	NA	NA	scRNA-seq	Characterized dormant malignant plasma cells expressing a unique myeloid signature; AXL gene retains myeloma cells in a dormant state
Zavidij et al. [39]	Immune cells of BM from healthy donors (*n* = 9), MGUS (*n* = 5), low-risk SMM (*n* = 3), high-risk SMM (*n* = 8), NDMM (*n* = 7)	32	scRNA-seq	Revealed early alterations in immune cell composition and features associated with MM progression
Liu et al. [37]	Tumor cells and immune cells from paired SMM-NDMM (*n* = 3), paired NDMM-RRMM (*n* = 6), NDMM (*n* = 4), RRMM (*n* = 1)	14	scRNA-seq, WGS/ WES	Longitudinal multi-omic investigation of samples at different disease stages; integrated analysis identified 3 clonal patterns; detected PCs population with B-cell features suggesting PCs origin; targeted protein analyses confirmed differential expression among distinct clusters
Cohen et al. [131]	Tumor cells from healthy donors (*n* = 11), NDMM (*n* = 15), PRMM (*n* = 41)	67	scRNA-seq	Identified expression signatures of patients with primary refractory MM participating in clinical trials correlated with clinical outcomes; Identification of a signature of highly resistant MM and revealed PPIA as a promising therapeutic target
de Jong et al. [132]	Stromal cells, immune cells and tumor cells from healthy donors (*n* = 7), NDMM (*n* = 13)	20	scRNA-seq, Bulk RNA-seq	Identified inflammatory mesenchymal stromal cells population with myeloma-specific pattern in BME, which persisted in the BM even after successful antitumor treatment
Cho et al. [133]	BM and PB sorted NK cells from NDMM (*n* = 3)	3	scRNA-seq, Flow cytometry	Identified a subset of adaptive NK cells with distinct immunophenotypic features and exert substantial cytotoxic activity against myeloma cells in the presence of daratumumab
Ryu et al. [134]	BM and extramedullary biopsies of MM (*n* = 15)	15	scRNA-seq	Aggressive extramedullary myeloma is associated with transcriptional signatures that support myeloma cell growth and immune evasion
He et al. [34]	BM CD138^+^ cells from NDMM (*n* = 12) and RRMM (*n* = 6)	18	scRNA-seq, scVDJ-seq	Revealed that myeloma cells from most of NDMM and RRMM patients consist of a major clone, and identified several meta-programs and biomarkers correlated with disease progression and relapse of MM
Frede et al. [36]	Sorted CD138^+^ and CD45^+^ cells from healthy donors (*n* = 2) and RRMM (*n* = 8)	10	scRNA-seq, scATAC-seq	Revealed profound intratumoral heterogeneity and a loss of lineage restriction in myeloma cells from RRMM. Anti-myeloma treatment reduced developmental potential and CXCR4 may serve as a promising target for immunotherapy
Li et al. [35]	BMMC and PBMC from healthy donors (*n* = 3) and MM during treatment (*n* = 10)	13	scRNA-seq, Flow cytometry	Identified a subset of ZNF683^+^ NK cells in MM patients and exhibited an exhausted phenotype by inhibiting EAT-2/SLAMF7 axis
Merz et al. [135]	BM and osteolytic lesion samples from NDMM (*n* = 7) and RRMM (*n* = 3)	10	scRNA-seq, WES	Spatially revealed enormous inter- and intra-tumor heterogeneity in both BM and osteolytic lesions, and identified key genes associated with myeloma bone disease
Tirier et al. [38]	Tumor and immune cells from RRMM (*n* = 20) during treatment	20	scRNA-seq	Demonstrated that 1q + subclones harbored a distinct transcriptional signature and showed frequent expansion during therapy. The BME was shaped towards an immune suppressive status in RRMM via increased expression of inflammatory cytokines and enhanced interaction with the myeloid cell subsets
Rodriguez-Marquez et al. [136]	anti-BCMA CAR-T cells from MM patients (*n* = 3)	3	scRNA-seq	Identified regulons involved with CAR^High^ T cells activation and exhaustion, revealing potential mechanisms underlying their differential functionality
Da Vià et al. [137]	anti-BCMA CAR-T cells from RRMM at baseline and relapse (*n* = 1)	1	scRNA-seq	Identified a selected clone with loss of BCMA acquired by homozygous deletion and provided insights into CAR-T resistance
Liang et al. [138]	BM aspirates from healthy donor (*n* = 1), NDMM (*n* = 3) and RRMM (*n* = 4)	8	scRNA-seq, Nanopore sequencing	Revealed two clonal evolution patterns that related to tumor origins and microenvironment reprogramming
Masuda et al. [139]	Serial BM aspirates from RRMM (*n* = 1)	1	scRNA-seq	Identified a distinct cell cluster which emerged during lenalidomide treatment and eliminated after PI treatment. Identified a drug-response gene; PELI2, which confers the PI sensitivity and serves as a predictive biomarker
Jung et al. [140]	BMMCs from NDMM (*n* = 18) at baseline and after treatment	18	scRNA-seq	Identified a 24-gene signature that upregulated in SORs, revealed dysfunctional phenotypes of T cells in SORs, identified three monocytes subsets associated with bortezomib responsiveness
Yao et al. [141]	BMMCs from NDMM (*n* = 18)	18	scRNA-seq, CyTOF, CITE-seq	CITE-seq showed advantages in distinguishing T-cell subtypes. CD4^+^ T/CD8^+^ T cells ratio showed a decrease in MM patients with ISS stage 3
Li et al. [142]	BMMCs from MGUS (*n* = 4), NDMM (*n* = 5) and RRMM (*n* = 5)	14	scRNA-seq, Flow cytometry, Bulk RNA-seq	Revealed alterations in MM-TME during disease progression and TAM reprogramming, targeting both CD47 and MIF showed potent anti-MM effects

*BM* Bone marrow, *PB* Peripheral blood, *MGUS* Monoclonal gammopathy of undetermined significance, *NDMM* Newly diagnosed multiple myeloma, *MM* Multiple myeloma, *SMM* Smoldering multiple myeloma, *WES* Whole exon sequencing, *CTCs* Circulating tumor cells, *BMPCs* Bone marrow plasma cells, *RRMM* Relapsed refractory multiple myeloma, *PCs* Plasma cells, *BME* Bone marrow microenvironment, *CAR-T* Chimeric antigen receptor-T, *PI* Proteasome inhibitor, *BMMC* Bone marrow mononuclear cell, *SORs* Suboptimal responders, *CyTOF* Cytometry by time of flight, *CITE-seq* Cellular indexing of transcriptomes and epitopes by sequencing, *ISS* International staging system, *TME* Tumor microenvironment, *TAM* Tumor-associated macrophage

### Understanding the heterogeneity of MM

MM is a clonal disease with strong inter- and intra-tumoral heterogeneity, and these heterogeneities are reflected genetically, epigenetically, and phenotypically [5, 7, 143]. Intra-tumoural heterogeneity occurs when different tumour cells within a patient display different genetic or phenotypic characteristics. On the other hand, inter-tumoural heterogeneity indicates genomic and biological variations across different MM individuals. In the search for potential target therapies, gene expression profiling is a widely used method to characterize tumor phenotype in individual patients, however, most cancers display intra-tumoral heterogeneity, which may have impacts on drug response to specific therapeutics and clinical outcomes. With SCS technologies, tumor heterogeneity in myeloma can be assessed at a single-cell resolution, enabling researchers to unravel the complex biological nature of MM.

In the initial studies of scRNA-seq, samples were taken from patients at multiple time points in order to trace the dynamic tumoral evolution. In an early single-cell whole-exome sequencing (WES) study of six patients with t(11;14) MM, it was found that there were two to six major subclones present at the time of the diagnosis of MM and the existence of clones with both linear and branching evolution patterns [144]. As a result of subsequent single-cell genomic efforts, SCS have been applied to pursued molecular profiling of MM and its premalignant stages, with higher resolution than conventional NGS method. Recently, Liu et al. analyzed 14 MM patients throughout the disease progression by multi-omic combining bulk DNA and scRNA sequencing, and identified three patterns of clonal evolution, including stability (from premalignancy to diagnosis), and gain or loss (from diagnosis to relapse) [37]. Notably, by mapping PCs together with B cell lineage, they found “B cell-like” plasma cell subpopulations that cluster closely with B cells. This study provides an important guide to delineate plasma/myeloma cells origin, and motivates us go deeper into investigating myeloma cells development and its implications in MM malignancy.

By examining scRNA-seq data from patients with MGUS, SMM, MM, and light chain amyloidosis, the authors found that myeloma heterogeneity persisted throughout these different disease stages [33]. In addition, a recent scRNA-seq study of plasma cells from 26 patients at different disease stages revealed comprehensive intratumor heterogeneity by capturing shared transcriptional programs across patients, and demonstrated that scRNA-seq is sensitive enough to detect latent cytogenetic alterations in patients at asymptomatic stages with low tumor burden [145]. These findings emphasize the feasibility of applying scRNA-seq to detect changes in BM myeloma cells earlier than methods implemented currently in the clinic. Apart from human myeloma studies, a model of disease progression in Vκ*MYC mice was subjected to longitudinal single-cell depiction, and revealed that tumors at early stage contained subclonal CNVs which persisted throughout the disease progression [146]. More importantly, researchers identified a tumoral program involving GCN2 stress response that progressively activated during myeloma progression, and indicated the potential role of GCN2 as a promising therapeutic target [146]. These studies together highlight the application of single-cell technologies to profile the genetic and transcriptomic heterogeneity of tumor cells, and emphasize the necessity for their combination in studies aimed to explore disease progression of MM.

Multiple relapses and drug resistance are hallmarks of MM [147]. In recent studies, scRNA-seq was performed to extensively dissect MM resistance in refractory/relapsed patients. Through single-cell transcriptomic and chromatin accessibility profiling, Frede et al. demonstrated coexisting transcriptional programs in single tumor cells of relapsed refractory MM (RRMM) [36]. They detected six distinct expression programs across RRMM patients including cell cycle, different cell signaling pathways, including KRAS-MAPK, IL-6-STAT3 and IL-2-STAT5 pathways, as well as the interferon response. More importantly, pseudotime trajectories showed that myeloma cells exhibited a higher developmental potential than normal plasma cells, suggesting their lineage infidelity and plasticity. They further demonstrated that standard treatment can reduce differential potential while increase regulon activity, and CXCR4 may represent an attractive therapeutic target, since clinical trials have already tested CXCR4 antagonist for relapsed myeloma [148] and use for other clinical applications [149]. In another study published recently, scRNA-seq and variable-diversity-joining regions-targeted sequencing (scVDJ-seq) were performed to showed the transcriptomic and clonal diversity in newly diagnosed MM (NDMM) and RRMM patients [34]. Similarly, they observed universal intra-tumoral heterogeneity after identifying 8 meta-programs covered diverse biological functions, including the cell cycle that have been identified previously [36]. Comparing to NDMM, RRMM-specific myeloma cell clusters showed upregulations of the expression signatures of several immature progenitors (e.g. MMP and CLP) and representative target genes including STMN1, TUBB, TYMS, TUBA1B and HMGN2, which may contribute to relapse risk and dismal overall survival (OS).

In MM, clonal PCs accumulate in the BM, resulting in bone destruction and focal lesions [143]. The number of focal lesions served as a prognostic factor due to its correlation with the disease progression [150]. BM samples are collected mainly from the posterior iliac crest or from the sternum. Limited by this invasive procedure, repeated samplings are restricted. Nonetheless, Rasche et al. elegantly revealed genomic heterogeneity spatially, which was found in most of the patients at both chromosomal and mutational levels [150]. For instance, del(17p) exhibited spatial variation in two out of six MM patients. Progression events are frequently restricted to focal events, however initiating events are uniformly spread. High-risk disease distribution can be heterogeneous, and taking a "snapshot" at diagnosis at only one locus misses important information [151, 152]. Despite limited evidence of spatial heterogeneity from whole-exome sequencing, a recent report used scRNA-seq might provide potential mechanisms by which myeloma cells induce osteolytic lesions (OL) in one region, whereas other regions show no indications of bone destruction despite subtotal infiltration. Beyond the characterization of inter- and intra-tumor heterogeneity, the study identified OL-specific differentially expressed genes like DKK1, HGF and TIMP-1 compared to PCs from BM, and contributed to the understanding of mechanisms underlying myeloma bone disease [135].

Collectively, by integrated analysis of single-cell sequencing, these studies elucidated multiple layers of complexity regarding myeloma heterogeneity, including inter- and intra-patient heterogeneity across disease progression from precursor stages to active MM and relapse status, as well as additional spatial assessment in bone lesions. These data have proved fruitfully in resolving intricate molecular dynamics of MM pathogenesis and disease relapse by SCS technologies.

### Uncovering the mechanisms of drug resistance

The main goals of anti-myeloma therapy are to achieve greater depth of response and improve outcome of MM patients. Drug-induced changes possess the potential to reveal important insights into the molecular mechanisms in action of chemotherapeutics. A better understanding of the specific molecular signatures changed during the course of treatment is key to understand the detailed mechanisms behind drug response or resistance. With the capacity to investigate tumour-intrinsic transcriptome profiles, and tumour heterogeneity, scRNA-seq analysis combined with dynamic sampling and detailed genetic profiling could largely support the identification of drug-response genes and the understanding of drug-induced changes in the clonal dynamics. A recent scRNA-seq analysis of BM cells derived from 20 RRMM patients revealed that MM consists of diverse genetic clones with marked transcriptional changes and clonal dynamics before and after treatment [38]. Gain/amplification of chromosome 1q (1q +) region has been shown to be associated with poor outcomes of patients with MM [153, 154]. Using scRNA-seq tool, Tirier et al. detected rare subclones harboring 1q + that comprised ~ 2% of the cells, and these cells cannot be separated into a certain transcriptional cluster, demonstrating the sensitivity of SCS in detecting rare subclones in MM [38]. In clone dynamics during treatment in RRMM, they found that 1q + clones often showed expansion or remained stable and therefore displayed a compelling robustness anti-myeloma drug in RRMM. Interestingly, by screening the upregulated genes located in 1q, they found 18 genes were exclusively detected by scRNA-seq, which were not found in bulk RNA-seq, and these genes were associated with various biological processes, such as apoptosis, proteasome, and signaling from BME, which may explain mechanisms of drug resistance in different treatment strategies. Additionally, the 1q + specific tumor program can be used to faithfully detected 1q + subclones with low abundant before treatment, which could be exploited in other scRNA-seq data sets of MM.

Longitudinal monitoring during treatment and remission allowed the researchers to evaluate disease status, drug response and provide real-time notification of clonal emergence or reduction for clinicians. ScRNA-seq analysis can be incorporated with longitudinal sampling and intensive genetic and transcriptional profiling, which would bring a deeper understanding of treatment-induced alterations in the clonal dynamics and identification of drug-response genes. Masuda et al. performed scRNA-seq on MM cells from a patient with relapsed MM by temporal sampling during multiple anti-MM drugs [139]. Although only one sample was assessed, they identified a distinct cell cluster which arose after lenalidomide treatment and eliminated after PI treatment. Further investigation identified a drug-responsive gene, PELI2, which may confer PI sensitivity, and in vitro experiments and computational analysis further confirmed that PELI2 presents a predictive biomarker of the PI responsiveness in MM patients. This study highlights the advantages of SCS in detecting myeloma cell population with low abundance during therapy and examining drug-induced cellular changes. These findings warrant further research on more MM patients by utilizing SCS approaches. In another recent study assessing drug response in a larger sample size, scRNA-seq data were collected from optimal responders (ORs) who achieved the best response in CR or VGPR and suboptimal responders (SORs) with best response of PR, MR, SD, or PD. By comparing ORs with SORs, the authors identified a 24-gene signature that upregulated in SORs, and further validated its association with bortezomib responsiveness and poor prognosis [140]. Additionally, our recent study that performed scRNA-seq on MM samples during anti-myeloma treatment, revealed dynamic changes in both tumoral and microenvironmental programs, and highlighted the SCS derived-benefits for comprehensively understanding overall and detailed features in myeloma BM ecosystems during therapy [155].

Based on the capability of predicting drug response, scRNA-seq can be used in clinical trials to predict resistance and guide therapy development for resensitizing tumors to treatment [131]. In this study, the authors applied massively parallel RNA SCS framework (MARS-Seq) on BM samples from a cohort of 41 individuals with primary refractory MM (PRMM), who experienced primary resistance or early relapse after a first-line bortezomib-based agent [131]. These patients were enrolled in the KYDAR trial and treated with a combination agent: daratumumab, carfilzomib, lenalidomide, and dexamethasone (DARA-KRD). Through exploring drug resistant mechanism, the authors found 66 differential expressed genes that could be clustered into 3 modules with capacity to distinguish PRMM from NDMM, and integrating both ≥ double hit and module 1-high in multivariate analyses was found to increase the prediction power of examining patients’ outcomes. Moreover, by comparing responders and non-responders during DARA-KRD treatment, they generated a resistance signature, which contained substantial overlap genes with module 1 and suggested that this newly discovered resistant signatures may be relevant throughout the course of several lines of therapy. Clinical utility of these signatures might facilitate to predict the risk of drug resistance in a group of MM patients. This approach could be implemented using routine methods such as qPCR and also by machine learning-based method. Another implication of this study is that by combining longitudinal scRNA-seq and clonal analysis using B-cell receptor sequencing (BCR-seq), they were able to score a single clone in every tumor and detected small resistant clones that appeared during treatment. Thus, this study presents a framework for developing a drug-resistance atlas that could revolutionize clinical practice in the future.

### Dissecting bone marrow microenvironment

The interplay between myeloma cells and the BME is crucial for tumor development, treatment and disease progression [11, 156–158]. Several cell types in BME including stromal cells [159, 160], osteoblasts [156, 161], osteoclasts [161], immune cells [13, 162, 163] (e.g., B cells, T cells, natural killer cells, monocytes, myeloid-derived suppressor cells) and endothelial cells [156, 164] present in the BM and generate a unique milieu that favors MM cells immune evasion, bone disease and promotes drug resistance. Reciprocal interactions between the various compartments in BME and the MM cells are essential to regulate differentiation, migration, growth and survival of the malignant plasma cells. In-depth understanding of the tumour ecosystem and cell-cell interactions could faithfully provide insights for developing novel and more effective therapeutics for MM patients. One limitation of traditional genomic studies is that they are focused on the myeloma cells only and thus ignore the complex interactions with microenvironment. Single-cell technology is a powerful approach to investigate the sophisticated TME and can be exploited for precision medicine and overcoming treatment resistance. Here, we briefly introduce several key studies performing SCS approaches to explore TME in MM.

### T cells

T cells play central roles in anti-tumor immunity. In recent years, a variety of reports have revealed quantitative and functional T cell abnormalities in MM patients [165–168]. Early study on T cell subsets revealed three major abnormalities of T cells in myeloma, including (1) the emergence of cytotoxic T cell harboring the senescence-associated secretory phenotype (SASP) [166, 167]; (2) increase in the ratio between the regulatory T (Treg) cell and T helper 17 (Th17) [169, 170]. (3) the acquired Treg leading to immunosuppression [169, 171]. Single-cell profiling has allowed high-resolution mapping and comprehensive analysis of T cell heterogeneity and function. In an early study of MM that utilized scRNA-seq on patient-derived BME samples (CD138^−^ or CD45^+^ cell populations) from patients with MGUS, low-risk or high-risk SMM, and active MM, the authors revealed early alterations in immune cell composition at the precursor stages, with substantial T cells enrichment in particular [39]. This study demonstrated a shift from the manifestation of less-mature cytotoxic memory T cells with high GZMK expressing in non-malignant and MGUS patients, to an enrichment in more-mature cytotoxic memory T cells with GZMB expression at the SMM and MM stages. Later in vivo experiments using C57BL/KaLwRij mice with 5TGM1 cells injection revealed that this transition may reflect a mechanism that drives immune evasion during disease progression of MM. In regard to dissect T cell atlas and function in precursor stages, another SCS study of T cells from MGUS, SMM to active MM was conducted [172]. To increase the sample size, the authors combined their in-house data with samples from a published SCS data set [39] to perform a meta-analysis. They found that the anti-tumor immune response showed a decline from MGUS, SMM to MM, evident by a general decrease in naïve and memory CD4^+^ T cells and an increase of Treg together with CD8^+^ Eff 2 cells with an inhibitory phenotype. These two single-cell studies jointly demonstrated that early alterations of T cell populations have arisen in asymptomatic conditions of MGUS and SMM stages [39, 172]. Identifying the early immune events that promote MM progression by single-cell technologies will help to stratify patients based on their risk of progression, and offer therapeutic opportunities for early intervention.

Except for these quantitative changes, MM patients also exhibited relevant defects of T cell functions. Early studies showed that under the influence of TGF-β, released by regulatory immune cells and myeloma cells, T cells presented a remarkable reduction in IL-2-mediated autocrine proliferation [167], and diverse signaling impairment, including the downregulation of CD28, CD152, p56lck, ZAP-70, and PI3K, which were found in both CD4^+^ and CD8^+^ T cells in MM patients with advanced-stage [173]. A combined analysis of scRNA-seq and mass cytometric assay on immune cells in the MGUS and MM showed that during disease progression, T cell clusters with stem-like and tissue-resident signature, were depleted during MGUS-to-MM progression [174]. In particular, more substantial T cell exhaustion and senescence at the tumor site (BM) were observed than peripheral blood (PB) by expressing multiple molecules related to exhaustion (PD-1, CD160, 2B4, CTLA-4) and senescence (CD57, lack of CD28) [166]. Additionally, these T cells displayed reduced proliferative capability, defective cytotoxicity, and disability in IFN-γ production after antigen stimulation. In another report with in-depth immune checkpoints phenotyping of BM T cells in MM using mass cytometry-based single-cell analysis, Wang et al. confirmed higher expression of these conventional immune checkpoints in T cells and ligands in myeloma cells from MM patients compared with healthy donors (HDs), as well as several newly identified immune checkpoints including LAG3, Tim-3, and TIGIT [175]. Notably, a recent scRNA-seq study refined the molecular features of T cell by comparing MM patients with different bortezomib treatment responses [140], and the authors found that dysfunctional T cells expressing exhausting genes (PD-1, TIGIT, TIM3, CD244, LAG3, CTLA4 and TOX) were enriched in SORs compared to the ORs patients. Moreover, an upregulation of LAG3, KLRG1, CD47 and IFNG which have been previously linked to dysfunctional T cells in RRMM patients, pointing to the key roles of T cell dysfunction in drug resistance. In the immune cell populations that play roles in the tumor microenvironment, innate-like T cells expressing T cell receptors composed of γ and δ chains (γδ T cells) are of particular interest, and previous reports have demonstrated their impaired immune function during MM disease progression [176]. Interestingly, Tirier et al. showed that among all T cell populations, the highest expression of the exhaustion signature was detected in γδ T cells of RRMM patients by upregulating expressions of multiple inhibitory receptors, including LAG3, KLRG1, VSIR and TIGIT [38]. And in some patients, this exhaustion signature showed increased after treatment. By exerting antigen-driven cytotoxicity against target cells in an MHC-independent fashion, γδ T cells can recognize a broad variety of antigens to exert their potent and broad anti-tumour activity [177]. The findings of exhaustion of γδ T in RRMM and other infiltrated tumor tissues [178–180] suggest that these cells have a similar ability to respond to immune checkpoint inhibitors (ICIs) as traditional effector T cells. Thus, we can expect that reinvigorating γδ T cells by targeting inhibitory molecules might be beneficial for developing novel effective immunotherapies.

Recent studies have illustrated that defective metabolic flexibility related to cancer cells can lead to an inefficacy in anti-tumor immune response and linked to cancer progression [181–183]. The impaired energetic metabolism also involved with the myeloma oncogenesis and clinical outcomes of MM patients [184]. In a recent study, Lv et al. performed scRNA-seq on immune cells from NDMM and HD, and found that CD8^+^ T effector cells and natural killing (NK) cells with high tumor infiltration exhibited distinct metabolic features with comparison to those with low tumor infiltration and HD [185]. The abnormal metabolism was reflected by impaired metabolism amino acid including arginine, proline, glycine, serine metabolism and enhanced glycolysis/gluconeogenesis, oxidative phosphorylation and lipid metabolism in CD8-XCL2 memory T cells and CD8-GNLY effector T cells. The finding of defective metabolism was dependent on pathway analysis, further functional investigation would be needed to confirm the observations and dissect the detailed underlying mechanisms. The authors also revealed an upregulation of PIM kinases in effector CD8 T cells, supporting that targeting PIM kinases served as a feasible strategy to restore the immune cells function through metabolism regulation.

Chimeric antigen receptor (CAR) T-cell therapies have achieved impressive efficacy in MM but also bring its own set of challenges including durability of CAR-T cells, toxicities and therapeutic resistance [186–188]. A recent study performed single-cell genomic on temporal samples from a MM patient who relapsed after initial anti-BCMA CAR-T cell treatment [137]. The authors reported a clone selection with acquired deletion of BCMA, which resulted in lack of CAR-T cell proliferation following the second infusion, supporting the notion that antigen escape is one of the possible causes of CAR-T therapy failure [186, 189]. Interestingly, a recent study demonstrated that CAR level played a critical role in CAR-T cell activity with notable influence on clinical response [136]. To better understand the heterogeneity of CAR-T cells and the influence of CAR density on the transcriptional profile, the authors performed scRNA-seq and identified 23 clusters of CAR-T cell populations and observed that CAR^High^ T cells mainly localized within activated CD4^+^ cells. Furthermore, in the CD8^+^ T cell subset, CAR^High^ T cells showed a pre-exhausted feature. They then demonstrated that CAR density was associated with differential activation of regulatory networks, evident by activation of transcription factors NR4A1 and MAF, low active SATB1 regulon in CAR^High^ T cells, which could explain their exhausted phenotype. Therefore, single-cell omics enables the high-resolution molecular characterization of the activation and functional states of T cells, thus holding great potential for understanding CAR-T cell behavior. Given the very active clinical research on CAR-T cells in MM ongoing currently [188, 190, 191], we expect that in the near future, SCS could become a standard tool for clinical monitoring cell states and developing more effective CAR-T cell therapies.

### NK cells

NK cells have been identified as the primary effector cells of innate immunity [192–194]. Numerous studies have demonstrated the significance roles of NK cells in killing cancer cells or in tumor progression regulation [193, 195, 196]. Impairments of NK cell maturation, chemotaxis, cytokine production, and expression of effector molecules have been described in the context of MM [197, 198]. Therefore, restoring or enhancing the cytotoxicity of NK cells for MM treatment has become one of the key topics in recent years. In the first scRNA-seq study analyzing the alterations in immune microenvironment along MM progression, the authors identified 3 major NK subpopulations including CXCR4^+^ NK, CX3CR1^+^ NK and a less frequent CD56^bright^ NK population, and showed that NK cell proportion is frequently increased in the precursor stages indicating an early onset of an immune response [39]. Another recent study applied scRNA-seq to profile CD56^+^ NK cells from NDMM BM samples, have also acknowledged the CD56^bright^ NK population [133]. They further revealed three novel NK cell populations at single-cell level: adaptive NK-cell cluster characterized by high expression of KLRC2 and low expression of FCER1G, the terminal NK cluster expressed ZEB2 and B3GAT1 genes, a NK-HSP cluster characterized by the high expression of genes for heat shock protein (HSP), which was considered as stressed NK cells due to sample freezing and thawing. The uncovered population of adaptive NK was also identified in a recent study utilized scRNA-seq to dissect TME of MM patients receiving bortezomib-based treatment [140]. Their further investigation demonstrated that unlike conventional NK cells, adaptive NK cells displayed distinct immunophenotypic features with low expression of inhibitory receptors such as killer cell immunoglobulin like receptor (KIR), TIGIT and NKG2A. More importantly, with daratumumab treatment, adaptive NK cells from NDMM patients exhibited enhanced cytokine production and increased degranulation compared with conventional NK cells, suggesting that adaptive NK cells serve as an important mediator of antibody-dependent cellular cytotoxicity (ADCC) in MM.

Through conventional measuring methods, such as flow cytometry and bulk RNA-seq, the exhausted phenotype of NK cells in MM has been described in previous studies, reflected by expressions of immunosuppressive molecules, such as PD-1 [199, 200]. However, the detailed mechanisms driving NK exhaustion in myeloma have not been completely elucidated. Innovative studies are needed to dissect molecular mechanisms involving NK cell dysfunction. In order to characterize potential mediators of NK-cell exhaustion, our previous study performed single-cell transcriptome profiling on NK cells from MM patients and HD, and identified a distinct NK subset that enriched in MM patients [35]. This MM-specific NK cluster exhibited exhaustion phenotypes by upregulating inhibitory receptors including LAG3, KLRG1, and KIR. More importantly, we further identified a vital transcription factor ZNF683 that might be responsible for the NK-cell exhaustion. Mechanistically, ZNF683 can directly suppress SH2D1B (EAT-2) expression, thus disrupting EAT-2/SLAMF7-mediated activating signals and eventually promoting NK cell exhaustion. Likewise, a ZNF683^+^ NK cell subset was also found in nonkeratinizing nasopharyngeal carcinoma exhibiting similar exhausted phenotypes with upregulated LAG3 and TIGIT expressions [201]. These consistent findings lead to a confirmation of ZNF683^+^ NK cell present in cancer context and highlight potential roles of ZNF683 in regulating NK cell function. These findings were uncovered mainly based on scRNA-seq profiling on NK cells in both studies, and we believe it may largely rely on the sensitivity of single-cell technology in capturing low abundant cells that conventional methods cannot provide.

### Monocytes/macrophages

Among the most important regulators of inflammation, monocytes and macrophages play important roles in cancer-associated inflammation [202, 203]. Their critical roles in tumor progression have been extensively described in solid tumors and hematological malignancies, including MM [204, 205]. Indeed, it has been proposed that within the BME, tumor-associated monocytes and macrophages (TAMs) are able to protect MM cells from treatment-induced apoptosis, promote angiogenesis and immune evasion [206]. In addition, several studies confirmed the roles of TAM in contributing resistance to common anti-MM regimens such as melphalan or bortezomib [207, 208].

Currently, a number of surface markers have been used to characterize TAMs (including CD14, CD68, CD163 and CD206), and two major functional macrophage states have been identified: M1 (inflammatory or “classically activated”), activating during infections and M2 (suppressive, “alternative pathway”) involving in wound healing and angiogenesis. Notably, M1 and M2 macrophages should be considered as two extremes instead of a continuum, however TAMs often exhibit a mixed transcriptional profile [209–211]. Consistently, a recent study applied scRNA-seq to dissect TME during disease progression of myeloma, and found that reprogrammed TAMs displayed a mixed phenotype with both M1 and M2 features. They also identified two TAM clusters exclusively emerged in the MM stage and exhibited higher M2 scores, suggesting higher pro-myeloma activity of these TAMs clusters [142]. Besides, a similar polarization pattern has also been observed in monocytes. In particular, three major subsets are recognized including classical (CD14^+^CD16^−^), intermediate (CD14^+^/^−^ CD16^low^), and non-classical monocytes (CD14^−^CD16^+^), and the latter subset is considered to harbor a tumor-promoting phenotype [212]. In the absence of standard detection tool, the TAMs percentage within BM of MM patients has been found to be highly variable (ranging from near 0 up to 25%). TAMs frequency displays an increase during the development from MGUS to MM, and patients with a high CD163^+^ and CD206^+^ TAM infiltration are associated with worse prognosis [205, 213]. A recent scRNA-seq study reported that even though reduced in MGUS compared to advanced stages, mature monocytes/macrophages are already impaired, presenting a phenotypic transition resulting in the loss of MHC class II surface representation affecting their antigen-presenting capability [39]. Another scRNA-seq study on RRMM patient samples also revealed 3 distinct TAM clusters, in which TAM cluster 3 showed a unique profile with specific expression of immunosuppressive genes (e.g., CD84 and VSIG4) [38]. Additional immune cell interaction networks showed that TAMs presented as nodes of high connectivity with other immune cells in TME, and IL18R1/IL18RAP was identified as an immunosuppressive interaction between TAM3 and NK^bright^ cells [38].

### Dendritic cells

Dendritic cells (DCs) are vital antigen-presenting cells (APCs), which serve as a bridge between innate and adaptive immunity in response to various pathogens [214]. These cells can be distinguished into two subgroups based on their function: conventional DC (cDC) and plasmacytoid DC (pDC). DCs have been found playing crucial roles in the pathogenesis and disease progression of MM [215, 216]. There is a significant difference between MM patients and HDs, with a near 50% reduction in myeloid DCs and pDCs in PB [216, 217]. Interestingly, the evolution from MGUS to MM is linked to increase in both cDCs and pDCs from the BME. As a consequence of MM, DCs also display a significantly altered immunophenotypic profile [215]. The expression levels of CCR5, CCR7 and DEC205 down-regulated on DC subtypes in MM compared to those from healthy individuals [217]. Downregulation of CCR5 and CCR7 impairs DC migration to inflammation sites, whereas reduced DEC-205 expression dampens antigen uptake. Recent studies in transcriptomics have moved beyond expression arrays of bulk populations to single-cell profiling, helping to identify novel surface markers, reveal heterogeneity within subpopulations and to identify rare but crucial DC subclusters. A recent single-cell study identified four DC subclusters in BM samples from NDMM and HDs, in which cDC2 showed a reduction in MM patients compared with HD samples. And cDC1 population showed higher expressions of MHC I/II molecules and inflammatory cytokines and chemokines in MM with low tumor infiltration than MM with high tumor infiltration group [185]. These findings imply that antigen presentation still can be triggered by cDC1 in the context of low myeloma cell infiltration, but suppressed with the increased myeloma cell infiltration. Further pathway analysis suggested that the metabolic pattern of cDC1 was affected by high level of myeloma cells. pDC is a unique DC subset defined by its essential properties of secretory plasmacytoid morphology and abundant endoplasmic reticulum, and key roles in antiviral responses by producing type I interferon (IFN) [218]. This “non-canonical” DC subset have been reported to accumulate in the BM of MM patients, and these pDC failed to stimulate T-cell proliferation but supported malignant cell growth and survival by direct contact with myeloma cells [219]. Recently, Tirier et al. generated scRNA-seq profiles from 20 RRMM tumour samples to analyze the impacts of treatments on BME cell type abundance and phenotype [38]. They found pDCs showed an expansion upon IMiD-based treatment, as well as exhibited upregulation of inhibitory receptors including LGALS9, CLEC4A and CD300A in pDCs from RRMM. These data suggested that a remodeling pro-tumorigenic phenotype of pDC contributes to RRMM immunosuppression and resistance to IMiD-based treatment. Therefore, these studies confirm that targeting pDC-MM interplay offers a promising therapeutic strategy for overcoming drug resistance in MM.

Collectively, the DC impairment in MM leads to dysfunctional capability of antigen presentation and pro-tumor activity. Through improved understanding of the interactions between myeloma cell, cDC and pDC via single-cell methodologies, targeting the crosstalk between these cells may pave the way to successful DC-targeting immunotherapies in the future.

### Mesenchymal stromal cells

As an important component of the nonimmune microenvironment, mesenchymal stromal cell (MSC) has been reported to promote MM proliferation and induce drug resistance [160, 220]. Despite great enthusiasm in exploring the roles of MSCs in MM progression, the low frequency of MSCs in BM aspirates has hampered detailed investigations into the roles these cells. A recent scRNA-seq report conducted by de Jong et al. presents the first study using scRNA-seq to profile MSCs, as well as immune and myeloma cells, from patients with MM and healthy individuals to comprehensively dissect the microenvironment crosstalk in MM [132]. Using samples with over 200 million mononuclear cells and MSC enrichment allowed the authors to profile ~ 1,000-1,500 MSCs per patient. They identified total five MSC subsets, two of which (MSC1 and MSC2) were termed as iMSCs and were nearly exclusive to the MM samples. These cells were characterized by expressing inflammatory cytokines and chemokines, including several MSC regulators reported previously, such as IL6, CXCL8, CXCL2, PTGS2 and VEGFA, proteins involving in the tumor necrosis factor (TNF) pathway and CCL2, which has been shown to support myeloma cell migration via interacting with CCR2 [220–222]. Additionally, these iMSC subpopulations also expressed genes encoding CXCL5, CXCL3 and CD44, and the later one has been suggested as an iMSC marker in flow cytometry analysis. De Jong et al. demonstrated that IL-1β and TNF receptors were identified as key phenotypic mediators of iMSCs, which was further confirmed by the IL-1β and TNF induced activation of MSCs in vitro. The authors further investigated the intercellular crosstalk between MSC and immune or myeloma cells, and they found that TNF and IL-1β were mainly expressed by cytotoxic T cells, NK cells and monocytes respectively. Aside from communicating with myeloid subsets, iMSCs committed with proliferating MM cells via the CCL2-CCR2 signaling pathway. More importantly, they demonstrated that stromal inflammation in myeloma BME induced by immune cells persisted even achieving successful induction therapy, indicating a potential effect of iMSCs in myeloma relapse.

Taken together, high-throughput SCS technologies enhance the ability of researchers to comprehensively characterize the cellular heterogeneity, temporal/spatial evolution of tumor cells and infiltrated immune and stromal cells in MM. These approaches provide insights into the mechanisms of drug resistance, immune suppression, and disease relapse in individual MM patients, thereby contributing to develop more effective and personalized anti-myeloma therapeutics.

## Future perspectives and directions

Our understanding of myeloma biology has been improved dramatically, owing to the rapid development and application of single-cell technologies. Some remained fundamental questions in myeloma research can be further explored by SCS. First, MM is an extremely complex disease whose origin and inheritability remain controversial and require detailed investigation. To explore myeloma origin, a comprehensive strategy for the identification of MMSCs could be aided by advanced SCS techniques. Owing to its ability to capture rare cell population with high-purity, and to unravel initiating point along the differential trajectory, SCS holds promise to detected the MMSCs population and identify its real markers. Indeed, scRNA-seq have been utilized to unravel the hierarchies of tumors and their progenitor cancer stem cells (CSCs) in many cancer types [223–227]. In addition to application in deciphering tumor origin, scRNA-seq can also be used to characterize unknown features of CSC subtypes, such as its heterogeneity, unique stem/stem-like features [103, 228, 229], which will provide novel insights into the underlying mechanisms of CSC mediated self-renewal and drug resistance. Second, single-cell profiling of myeloma cells and the surrounding immune/stromal cells enables the characterization of the sophisticated BM ecosystem, specially the complex crosstalk inferred from scRNA-seq data via predicting interactions of ligand-receptor pairs between different cell subsets. Novel approaches are also being developed to allow the investigation of physical intercellular interactions, especially the spatially resolved scRNA-seq methods. These attempts will bring a better understanding of the intricately cellular interactions within the BME. Besides, single-cell T cell receptor sequencing (scTCR-seq), with the capability to track each T-cell clone through paired sequencing of the T-cell receptor genes, can be integrated with high-dimensional single-cell spatial analysis, which has become particularly relevant to immuno-oncology [29]. And combination of scRNA-seq and scTCR-seq offers an effective method to analyze the phenotypic and functional characteristics of immune cells during disease progression or over the course of treatment. Third, high throughput spatial technologies are particularly suitable for BM and extramedullary exploration of MM, which presents a spatially divergent disease with multi-region genomic heterogeneity [135, 150]. Combined with imaging modalities, spatial transcriptomic techniques will be able to add important information on cellular location within the bone niche, contributing to a better understanding of myeloma bone disease. Extramedullary disease (EMD) presents an aggressive form of MM, which is linked to high-risk genomic alterations, increased proliferation and poor survival [230]. Innovative technologies recently revealed a complex spatial architecture of solid tumors [231–234] marked by multicellular niches supporting tumor growth or preventing immune cell infiltration. However, whether these observations hold true for EMD is largely unknown. Thus, investigating the EMD microenvironment in a spatial context using spatial omics technologies will provide a deeper understanding of ecosystems outside the BM and underlying mechanisms involving extramedullary spread.

SCS technologies also offer powerful tools for unbiased discovery of novel drug targets for MM treatment. For example, Frede et al. showed that in RRMM patients, treatment induced significant changes in chromatin accessibility by scATAC-seq, and they identified a surface protein CXCR4 as an attractive candidate as surface markers that can be targeted by immunotherapy [36]. Furthermore, another study that paves a way for incorporating scRNA-seq in clinical trials, identified PPIA as a potent therapeutic target to overcome resistance [131]. A multi-omics tool called scDAb-seq was generated [235] by combining of scDNA-seq with surface protein mapping (AB-seq [236]), which enables the characterization of both genotypic and phenotypic features. Using this elegant assay, it’s possible to determine the proteogenomic profile of clones at specific stages. Additionally, this approach allows researchers to determine the vast cell-surface proteome or “surfaceome” that regulates direct or indirect cell-cell interactions, ligand-receptor induced signaling, presentation of MM-specific antigens for immunotherapy. Impressive progresses have been achieved on determining myeloma cell surface proteome and enable unbiased discovery of novel therapeutic targets [237, 238]. CyTOF offers a new cytometric method for deep profiling single-cell protein on myeloma cells [37, 239] and immune populations [141, 240, 241]. Given the great success in immunotherapy in myeloma, we believe that application of single-cell proteomic sequencing will revolutionize the research in cancer pathology, especially when combined with multi-omic platforms, such as genetic and transcriptomic data at a single-cell resolution, and more importantly, dramatically contribute to identify novel targets for MM immunotherapy.

Preclinical models including human-derived cell lines [242] and mouse models [243] represent solid platforms for drug testing and investigation of disease mechanisms. Owing to technological advances, single-cell omics have been employed in numerous studies using clinical samples and preclinical models to investigate various pathologic conditions [244–247]. Inter-patient heterogeneity remains a key barrier to transform the preclinical findings into the clinic, with some of the patients failed to benefit from new drugs in clinical trials. With the power to dissect tumor heterogeneity, SCS can be applied in different model systems and provides rich information related to genomic, phenotypic heterogeneity and tumor evolution, thus promoting precision medicine. In the condition of MM with genetical diversity, it’s hard to recapitulate all the clinical queries by preclinical models. A recent study represents an excellent example of preclinical research by establishing fifteen genetically engineered mouse models that covered the keys factors during MM pathogenesis, including diverse genetic heterogeneity, disease progression and BM microenvironment changes [248]. Their integrative results from coupled scRNA-seq and TCR-seq highlighted the values of applying high throughput SCS on mouse and patient samples to test and predict response to immunotherapy drug combinations. Therefore, with innovations in unprecedented resolution and unbiased insights from SCS, preclinical models will accelerate our ability to answer fundamental and pivotal questions from bench to bedside, and to reproducibly apply these technologies across cancer patient populations.

Beyond being exploratory tools, SCS technologies can undoubtedly achieve more values when translated and adopted into clinical practice. Regarding the clinical utility, in our view, SCS assessments should be conducted based on the clinical needs, thus help to resolve problems encountered in the clinic. For instance, accurately monitoring measurable residual disease (MRD), identifying high risk patients and overcoming drug resistance represent three of the major unmet medical needs in MM. For the MRD measurement, a recent study has developed a single-cell MRD (scMRD) assay by combining flow cytometric enrichment of the targeted precursor/blast population in AML samples with integrated scDNA-seq and immunophenotyping, and achieved significantly high sensitivity [249]. This attempt encourages us to test single-cell based MRD monitoring on MM patients, which would be particularly helpful in disease monitoring. High-risk MM (HRMM) patients have been shown to have poor prognosis, thus accurately identifying high-risk features is a critical issue in MM. Genomic characterization of functional HRMM patients was proposed in a recent report [250], suggesting that factors outside of the myeloma cells may be critical for us to recognize the real HRMM. Thus, we can expect that comprehensively profiling both tumor cells and immune/stromal cells by single-cell approaches holds a great potential for this purpose. RRMM patients remain hard to treat, mainly due to drug resistance. The great power in predicting drug resistance in MM patients by scRNA-seq has been shown in a recent multicenter clinical trial [131], which offers a blueprint for applying SCS in clinical trials to build a drug-resistance atlas, and to promote biomarkers discovery for novel therapeutics [251].

However, applications of current SCS approaches outlined in this review are confronting with costs and technological challenges, which impeding our next step on the road to the clinic. By far the most expensive part of SCS is the sequencing itself. With the rapid advancements in sequencing technologies and exploitation of cost-reduction strategies, we believe that the sequencing costs will continue to reduce. Besides, there are several important frameworks and infrastructure are required for clinical use, including: (1) introducing standardized analytic pipelines and quality-controlled workflows when dealing with large amount of SCS data. In particular, batch effects should be carefully examined and corrected to bring reliable clinical results; (2) controlling false positive or negative rates. ScRNA-seq and scDNA-seq data would generate false negative or positive results due to the data sparsity [252], coverage nonuniformity and allelic dropout events [253]. Thus, it’s necessary to select computational methods to reduce potential false-positive/negative errors. (3) establishing clear biospecimen collection and processing procedures based on the experimental design and goals, such as what types and formats (fresh, snap-frozen and/or FFPE) of samples should be collected, optimal timepoints to collect and how sample materials should be stored; (4) developing devices or systems that automate these SCS technologies and are compatible with existing clinical laboratory workflows, thus saving time and reducing labor efforts; (5) specializing core facilities that enable secure big-data storage and enhancing collaboration between clinicians and translational scientists; Together, with the rapid advancements and further technical improvements of SCS technologies, we will be able to make single-cell assessments feasible for real-world applications in the coming years.

## Conclusion

In closing, SCS technologies have already revolutionized many aspects of cancer translational research and are prepared to have a greater impact in clinic. As the last decade has seen NGS technologies transform modern oncology, we believe that single-cell methodologies will influence many areas of MM medicine in the same way, and will become a powerful tool that can be implemented for clinical practice. Over the coming years, emerging technologies such as spatial SCS and multi-omics approaches will further expand their utility in myeloma research, and bring benefits for MM patients on personalized medicine and precision prevention. Overall, we expect that the implementation of single-cell technologies in myeloma medicine over the next decade will bring huge improvements in the clinical translation and treatment of MM patients.

## Data Availability

Not applicable.

## Abbreviations

SCS: Single-cell sequencing
MM: Multiple myeloma
BM: Bone marrow
PIs: Proteasome inhibitors
IMiDs: Immunomodulatory drugs
BME: Bone marrow microenvironment
NGS: Next generation sequencing
CNVs: Copy number variations
WGA: Whole-genome amplification
DOP-PCR: Degenerate oligonucleotide primed polymerase chain reaction
MDA: Multiple displacement amplification
MALBAC: Multiple annealing and looping based amplification cycles
BMSCs: Bone marrow stromal cells
TME: Tumor microenvironment
MMSCs: Multiple myeloma stem cells
SP: Side population
scRNA-seq: Single-cell RNA sequencing
SNVs: Single nucleotide variants
UMIs: Unique molecular identifiers
PDAC: Pancreatic ductal adenocarcinomas
LINNAEUS: Lineage tracing by nuclease-activated editing of ubiquitous sequences
scDNA-seq: Single-cell DNA sequencing
ssRRBS: Single-cell reduced-representation bisulfite sequencing
ChIP-seq: Chromatin immunoprecipitation sequencing
scChIP-seq: Single-cell chromatin immunoprecipitation followed by sequencing
ATAC-seq: Assay for transposase-accessible chromatin by sequencing
scATAC-seq: Single-cell assay for transposase-accessible chromatin by sequencing
T-ATAC-seq: Transcript-indexed assay for transposase-accessible chromatin by sequencing
CUT&Tag: Cleavage under targets and tagmentation
IGS: In situ genome sequencing
MS: Mass spectrometry
CyTOF: Cytometry by time of flight
IMC: Imaging mass cytometry
LC-MS: Liquid chromatography-mass spectrometry
SCoPE-MS: Single-cell proteomics by mass spectrometry
scDam&T-seq: Single-cell DNA adenine methyltransferase identification and transcriptome sequencing
scM&T-Seq: Single-cell genome-wide methylome and transcriptome sequencing
CITE-seq: Cellular indexing of transcriptomes and epitopes by sequencing
REAP-seq: RNA expression and protein sequencing assay
RAID: Single-cell RNA and immuno-detection
WES: Whole-exome sequencing
MGUS: Monoclonal gammopathy of undetermined significance
SMM: Smoldering multiple myeloma
RRMM: Relapsed refractory multiple myeloma
IFN: Interferon
scVDJ-seq: Single-cell variable-diversity-joining regions-targeted sequencing
NDMM: Newly diagnosed multiple myeloma
MPP: Multipotent progenitor
CLP: Common lymphoid progenitor
OS: Overall survival
PCs: Plasma cells
OL: Osteolytic lesions
ORs: Optimal responders
SORs: Suboptimal responders
CR: Complete response
VGPR: Very good partial response
PR: Partial response
MR: Minimal response
SD: Stable disease
PD: Progressive disease
PRMM: Primary refractory multiple myeloma
SASP: Senescence-associated secretory phenotype
ICIs: Immune checkpoint inhibitors
CAR-T: Chimeric antigen receptor-T
HSP: Heat shock protein
KIR: Killer cell immunoglobulin like receptor
ADCC: Antibody-dependent cellular cytotoxicity
TF: Transcription factor
TAMs: Tumor-associated macrophages
DC: Dendritic cell
APCs: Antigen-presenting cells
cDC: Conventional dendritic cell
pDC: Plasmacytoid dendritic cell
MSC: Mesenchymal stromal cell
TNF: Tumor necrosis factor
CSCs: Cancer stem cells
scTCR-seq: Single-cell T cell receptor sequencing
EMD: Extramedullary disease
MRD: Measurable residual disease
scMRD: Single-cell measurable residual disease
HRMM: High-risk multiple myeloma
FFPE: Formalin-fixed paraffin-embedded
